# Prescription pattern analysis for antibiotics in working-age workers diagnosed with common cold

**DOI:** 10.1038/s41598-021-02204-3

**Published:** 2021-11-22

**Authors:** Yasuhiro Araki, Kenji Momo, Takeo Yasu, Kohtaro Ono, Takeshi Uchikura, Masayoshi Koinuma, Tadanori Sasaki

**Affiliations:** 1 Department of Hospital Pharmaceutics, School of Pharmacy, Showa University, Hatanodai 1-5-8, Shinagawa-ku, Tokyo, 142-8555 Japan; 2 Department of Medicinal Therapy Research, Pharmaceutical Education and Research Center, Meiji Pharmaceutical University, 2-522-1, Kiyose, Tokyo, Noshio 204-8588 Japan; 3 Department of Pharmacy, Showa University Hospital, Hatanodai 1-5-8, Shinagawa-ku, Tokyo, 142-8666 Japan; 4 Faculty of Pharmaceutical Sciences, Teikyo Heisei Unversity, Nakano 4-21-2, Nakano-ku, Tokyo, 164-8530 Japan

**Keywords:** Health care, Public health, Epidemiology

## Abstract

Antimicrobial resistance is a major health concern. A primary cause is the inappropriate use of antimicrobials, particularly by patients with upper respiratory tract infection. However, baseline information for antibiotic use for common cold before being applied the National Action Plan on Antimicrobial Resistance in Japan is lacking. Here, we analyzed the inappropriate use of antibiotics in the working-age workers. We used large claims data from an annual health check-up for at least 5 consecutive years. Among 201,223 participants, we included 18,659 working-age workers who were diagnosed with common cold at a clinic/hospital. We calculated the proportion of patients with common cold who were prescribed antibiotics and analyzed predictive factors associated with antibiotics prescription. Antibiotics were prescribed to 49.2% (n = 9180) of patients diagnosed with common cold. In the logistic regression analysis, the group taking antibiotics was predominantly younger, male, without chronic diseases, and diagnosed at a small hospital/clinic (where the number of beds was 0–19). Cephems accounted for the highest proportion of prescribed antibiotics, with 40–45% of patients being prescribed antibiotics. Our data may be applied to prioritize resources such as medical staff-intervention or education of working-age people without chronic diseases who visit clinics for common cold to avoid the potential inappropriate use of antibiotics and prevent antimicrobial resistance acceleration.

## Introduction

In recent years, antimicrobial resistance (AMR) has become a major public health concern. In May 2015, the World Health Organization developed a global action plan for AMR^1^. A report on the socio-economic impact of AMR by the United Kingdom^2^ showed that AMR-related deaths are estimated to be 10 million per year by 2050. Moreover, the Centers for Disease Control and Prevention of the United States stated in a report issued in 2019^3^ that more than 2.8 million people in the United States are infected with drug-resistant microorganisms each year, resulting in more than 35,000 deaths.

One of the causes of AMR is the inappropriate use of antimicrobials. A total of 30% of antimicrobial prescriptions have been reported to be inappropriate^4^. In particular, treatment of upper respiratory tract infection accounts for approximately 50% of inappropriate prescription of antibiotics^5^. Teratani et al. retrospectively assessed antimicrobial drug selection and generation in acute respiratory tract infections (ARTIs) based on insurance claims data from 2013 to 2015 and reported that antibiotics are prescribed in more than 40% of outpatients diagnosed with ARTIs in Japan^6^. Traditionally, broad-spectrum antimicrobial agents are favored in Japan. A survey using insurance claims data from 2009 to 2013 found that 92.4% of the agents were oral antimicrobial agents with a high use of oral third-generation cephalosporins and fluoroquinolones, and it was noted that the use of macrolide antimicrobials was high^7^. In 2019, Tsuzuki et al. published the results of the first study on deaths owing to AMR in Japan^8^. This report estimated the number of deaths due to bloodstream infections (BSIs) caused by methicillin-resistant *Staphylococcus aureus* and fluoroquinolone-resistant *Escherichia coli* in Japan from 2011 to 2017, and death owing to BSI caused by those infections in 2017 was estimated at approximately 8000.

The National Action Plan on AMR^9^ was applied in April 2016 in Japan to avoid the inappropriate use of antibiotics. However, the baseline information for antibiotics before being applied the National Action Plan on AMR was lacking. In general, common cold is considered an infectious disease caused by viruses such as adenoviruses, rhinoviruses, and coronaviruses. Studies have shown that many symptoms of upper respiratory tract infections do not improve with antibacterial medication^10^. Contrarily, the use of antibiotics for common cold in adult participants has significantly greater risks of adverse reactions with antibiotics than with placebo (risk ratio 2.62; 95% confidence interval 1.32–5.18)^10^. In the US, antibiotics were implicated in 19.3% of all emergency department (ED) visits for drug-related adverse events, and a wide range of antibiotics was associated with ED visits. Minimizing unnecessary antimicrobial use could significantly reduce the direct risks of drug-related adverse events in individual patients.

However, because of misconceptions tendency regarding antibiotics in Japan, working-age workers likely to use inappropriately of antibiotics to avoid the absence of work^11^. Therefore, we used large claims data in the population of working-age workers to obtain baseline information before AMR application in working-age workers and clarify predictive factors for prescribing antibiotics for common cold.

## Results

### Primally endpoint for the proportion of antibiotics in common cold drugs

Among eligible patients, 49.2% of patients diagnosed with common cold were prescribed antibiotics (9180 [male/female: 7882/1298, 40.2 ± 9.9 years] vs. 9479 [male/female: 8015/1464, 42.6 ± 10.2 years]) (Table 1). The group prescribed antibiotics was younger (p < 0.001) and had a higher proportion of males (p = 0.012) than the group not prescribed antibiotics. The number of beds of the medical institution diagnosed with common cold in the group prescribed antibiotics was higher in the 0–19 beds but over 20 beds (p < 0.001). Most chronic diseases without chronic lower respiratory conditions were observed to be higher in the group prescribed antibiotics than in the group not prescribed antibiotics.

**Table 1:** Patients’ characteristics.

	With antibiotics	Without antibiotics	p-value
n (male/female)	7882/1298	8015/1464	0.012
Age [SD]	40.2 [9.9]	42.6 [10.2]	< 0.001
Number of beds (%)			
500-	181 [2.0]	503 [5.3]	< 0.001
300–499	221 [2.4]	598 [6.3]	< 0.001
200–299	119 [1.3]	263 [2.8]	< 0.001
100–199	228 [2.5]	497 [5.2]	< 0.001
20–99	149 [1.6]	333 [3.5]	< 0.001
0–19	8282 [90.2]	7285 [76.9]	< 0.001
Diseases (%)			
Neoplasms, C00-D49	282 [3.1]	428 [4.5]	< 0.001
Chronic lower respiratory diseases, J40-J47	1444 [15.7]	1322 [14.0]	< 0.001
Hypertensive diseases, I10-I15	790 [8.6]	1531 [16.2]	< 0.001
Atrial fibrillation and flutter, I48	30 [0.3]	66 [0.7]	< 0.001
Disorders of lipoprotein metabolism and other lipidemias, E78	901 [9.8]	1567 [16.5]	< 0.001
Inflammatory polyarthropathies, M05-M14	230 [2.5]	370 [3.9]	< 0.001
Diabetes mellitus, E10-14	442 [4.8]	848 [9.0]	< 0.001
Noninfective enteritis and colitis, K50-K52	56 [0.6]	116 [1.2]	< 0.001

**Figure 1:**
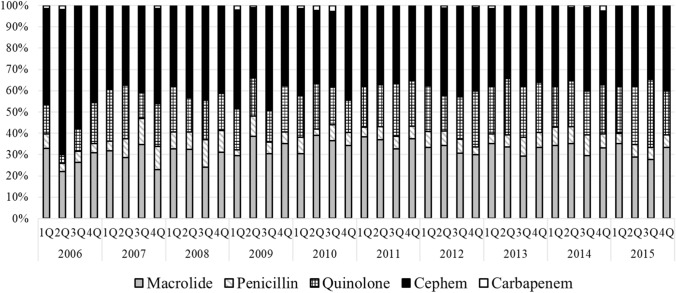
Trend for antibiotics class from 2006 to 2015 in patients with common cold.

The trend for antibiotic classes of macrolide, penicillin, quinolone, cephem, and carbapenem was described from 2006 to 2015 and divided quarterly that just before 10 years AMR initiation. (Fig. 1). No change was observed in the share of each antibiotic throughout the study period. In detail, the proportion of antibiotics was as follows: macrolides, 25–35%; penicillins, 5%; quinolones, 15–20%; cephems, 40–45%; and carbapenems, 0–1%.

### Secondary endpoint for predictive factors for prescribing antibiotics to patients diagnosed with common cold

In the logistic regression analysis, odds were lower for female patients than for male patients (0.86 [0.79–0.94], p = 0.0005) (Table 2). Additionally, odds for aging for + 1 years was negatively correlated with prescribed antibiotics in patients diagnosed with common cold (0.98 [0.98–0.99], p < 0.0001). The smaller the number of beds at the medical institution, the larger the odds of prescribing antibiotics to patients diagnosed with common cold. For example, as the reference for over 500 beds, odds observed in 100–199 beds were 1.21 [0.96–1.53], N.S. and 20–99 beds for 1.18 [0.91–1.53], N.S., but 0–19 beds for 2.90 [2.44–3.46], p < 0.0001. Regarding base comorbidities with hypertension, disorders of lipoprotein metabolism, and other lipidemias, inflammatory polyarthropathies , diabetes mellitus and noninfective enteritis and colitis was negatively correlated with the prescription of antibiotics. In contrast, in patients with chronic lower respiratory diseases, a positive correlation was observed for prescribing antibiotics.

**Table 2:** Predictive factors for prescribing antibiotics at the time for hospital/clinic visiting caused by common cold.

	Odds (95% CI)	p-value
Male	Reference	
Female	0.86 (0.79–0.94)	0.0005
Age, odds for + 1 year	0.98 (0.98–0.99)	< 0.0001
Number of beds		
500-	Reference	
300–499	0.92 (0.73–1.16)	0.4961
200–299	1.12 (0.85–1.48)	0.4229
100–199	1.21 (0.96–1.53)	0.1105
20–99	1.18 (0.91–1.53)	0.2175
0–19	2.90 (2.44–3.46)	< 0.0001
Diseases		
With neoplasms, C00-D49 (vs. without)	0.94 (0.80–1.11)	0.4660
With chronic lower respiratory diseases, J40-J47 (vs. without)	1.27 (1.17–1.38)	< 0.0001
With hypertensive diseases, I10-I15 (vs. without)	0.70 (0.63–0.77)	< 0.0001
With atrial fibrillation and flutter, I48 (vs. without)	0.81 (0.52–1.26)	0.3485
With disorders of lipoprotein metabolism and other lipidemias, E78 (vs. without)	0.81 (0.73–0.90)	< 0.0001
With inflammatory polyarthropathies, M05-M14 (vs. without)	0.83 (0.69–0.98)	0.0328
With diabetes mellitus, E10-14 (vs. without)	0.82 (0.72–0.94)	0.0038
With noninfective enteritis and colitis, K50-K52 (vs. without)	0.58 (0.42–0.81)	0.0013

## Discussion

In our study, we found that 49% of working-age workers diagnosed with common cold were prescribed antibiotics. Predictive factors for prescribing antibiotics were male sex, younger age, and chronic lower respiratory diseases. However, most of the baseline chronic diseases were negatively correlated with the prescription of antibiotics.

In this study, approximately 5% of patients received antibiotics with penicillin (Fig. 1). Amoxicillin is the only agent recommended in the Japanese guidelines^12^ when beta-hemolytic *Streptococcus* group A is detected by a culture test in patients with common cold. In the United States and European countries, the most prescribed antimicrobials for patients with colds are penicillins. For example, according to a survey of outpatient prescriptions in the United States, penicillins were the most prescribed antimicrobials in the outpatient setting (23%), followed by quinolones (20%) and macrolides (18%)^13^. A survey in the Netherlands reported that 39%, 30%, and 12% of antimicrobial prescriptions for upper respiratory tract indications were doxycycline, amoxicillin, and macrolides, respectively^5^. From a global perspective, Japan has lower penicillin use and higher macrolide or cephalosporine use than other countries. In our study, younger male workers of working age and without chronic disease had higher odds of recovering a prescription for antibiotics than their counterparts (Tables 1 and 2). The reasons for the prescription trend in Japan are different from those in other countries, but the environmental differences are partially explained by the differences in antibiotic prescription trends.

The medical environment in Japan is considered to be one of the reasons why antimicrobials are still widely prescribed for common cold patients, including those with ARTIs, in Japan. Expenditure for health care of per capita of Japan is middle in OECD countries, it costs $4822.8 (2019) per year, and it ranks 15th among 37 OECD countries^14^. Moreover, the out-pocket expenditure is ranked relatively low, i.e., 5th among OECD countries at 11.6% (2017)^14^. Because of the universal health insurance system and few restrictions on access to medical institutions, the annual number of outpatient consultations per capita is 12.6 (2017), the second highest among OECD countries after Korea^14^. It is assumed that there is an environment in which people visit medical institutions even for minor illnesses and more frequently than that observed in other OECD countries.

Despite the high level of medical use, knowledge of antimicrobials among the general adult population in Japan is not as high as that in Europe. Among 3390 respondents, only 741 (21.9%) correctly responded that antibiotics do not kill viruses, which is twice as high as the correct response rate in the EU (43%). Another 1376 (40.6%) respondents answered incorrectly that “antibiotics are effective against cold and flu.” Another 345 (10.2%) respondents wanted to prescribe antibiotics themselves and 1023 (30.2%) wanted their doctors to prescribe antibiotics^15^.

Various factors may explain why Japanese people believe that patients prefer prescribed antimicrobial agents for cold symptoms. Social background was suggested to be a factor in a survey of Japanese adults (20–39 years old; 208 valid responses) published in September 2007 by GlaxoSmithKline K.K., Japan^11^. Most respondents of the survey recognized that they were exposed to cold at work (75.0%) and during transportation (46.6%) and that it was difficult to take time off work for a cold (68.3%). This misperception that antimicrobials kill viruses combined with an environment and behavior that makes it easy for people to seek medical care even for minor illnesses and workers’ environments in which people are expected to return to work as soon as possible after catching a cold has led many people, especially working adults who think they have a cold, to ask their doctor for a prescription for antimicrobials in addition to cold medication, with doctors prescribing antimicrobials. This was one of the major problems associated with the inappropriate use of antibiotics. In addition, the rate of third-generation cephalosporin- and fluoroquinolone-resistant *E. coli* in isolated bacteria from patients is increasing in Japan^16^. Considering all these factors, third-generation cephalosporins and fluoroquinolones have not been administered to treat common cold symptoms in Japan.

Although the number of patients included in the survey was higher in Q1 and Q4 based on winter-seasonal variation, there were no significant seasonal differences in the types of antibiotics used. Overall, there were no notable differences between seasons in this survey, including other trends.

### Limitation

The scope of data collection for this study was before the issuance of official guidelines^12^ for the use of antimicrobials for patients with upper respiratory tract infections in Japan. Therefore, prescribing trends may have changed since March 2016, when the official guidelines were issued. In fact, Tsuzuki et al. reported that the annual additional cost of inappropriate antibiotic prescription for upper respiratory tract infections decreased from 2013 to 2016^17^. Career-pressure may higher in elderly working age population according to the social status; However, our data did not have the status for career. So, therefore, we analyzed age as the continuous variable, that display naturally differences in physical conditions.

## Conclusion

Our study shows the proportion of antibiotic prescriptions before AMR in working-age workers as a high-risk population for inappropriate antibiotic use in Japan. Our data may be applicable to before-and-after-analysis for AMR and predictive factors such as “0–19 beds of hospitals.” Moreover, “younger male workers” with antibiotic prescriptions need to be closely monitored and educated to avoid the inappropriate use of antibiotics when diagnosed with common cold.

Our data may be applied for prioritizing resources such as medical staff-intervention or education of working-age individuals without chronic diseases who visit clinics for common cold to avoid the potential inappropriate use of antibiotics and prevent the acceleration of AMR.

## Methods

### Data source

The JMDC claim database consists of anonymized data on > 3.6 million people (inpatients, outpatients, and pharmacy claims) aged ≤ 74 years from approximately 91.7% of all medical facilities (n = 90,021) in Japan as of 2016. All patients in the JMDC database have “social insurance” that includes working person and their families. As of August 2016, the database represented approximately 3.0% of the Japanese population.

### Study design and case identification flow

This study was based on the ethical guidelines for medical and health research involving human subjects in Japan. We did not obtain written or verbal informed consent because the claims data were fully anonymized before we accessed them.

**Figure 2:**
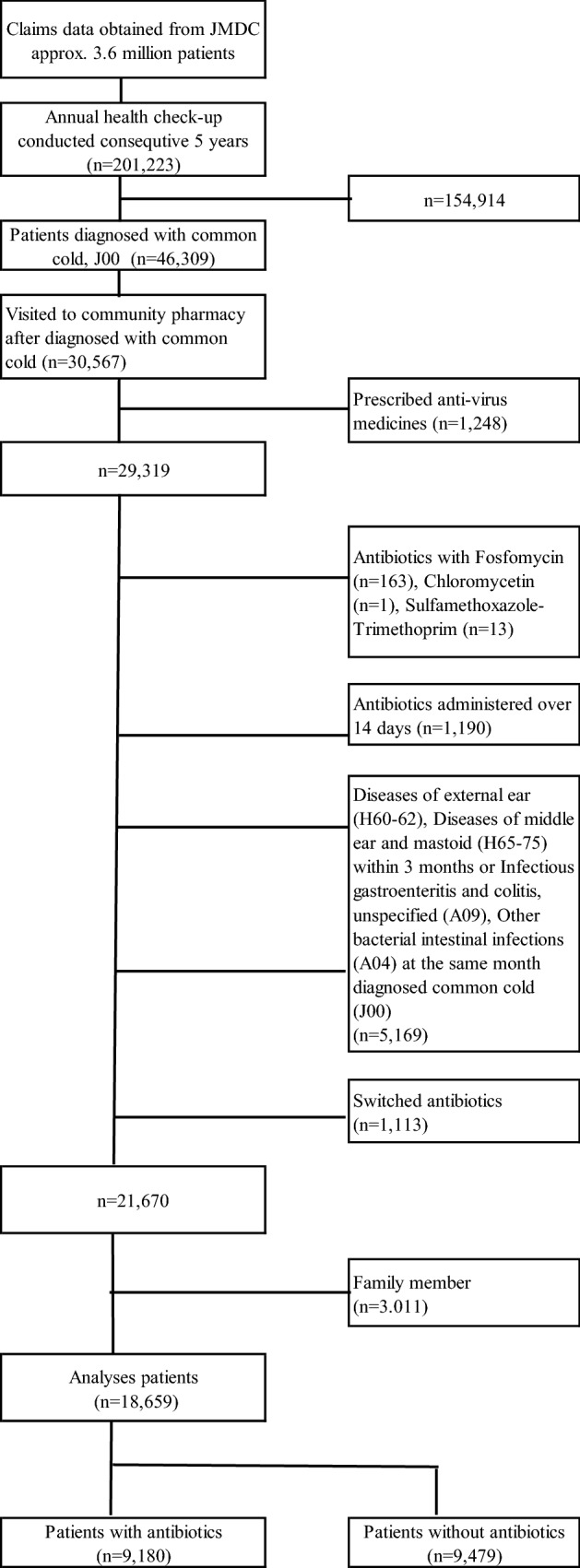
Patient identification flow.

This retrospective study involved 201,223 patients who underwent health check-ups between January 2005 and February 2016 for at least 5 consecutive years, and all participants were employees at a company, and their families (Fig. 2). Among those patients, 46,309 were diagnosed with common cold (ICD10: J00) more than once. In those patients, we analyzed prescriptions when patients were prescribed common cold drugs from a community pharmacy. If they were administered more than twice, we analyzed only the first diagnosis (n = 30,567). Among those patients, we excluded 1) those who were prescribed anti-viral drugs (n = 1248), because persons who are diagnosed with common cold but are prescribed anti-viral drugs may be influenza patients as well as common cold. It is known influenza can cause secondary bacterial pneumonia and it may need antibiotics. It is the reason why they were excluded from this study; 2) those who were prescribed antibiotics with fosfomycin (n = 163), chloromycetin (n = 1), and sulfamethoxazole-trimethoprim (n = 13), because we thought it was likely that all of those antibiotics were prescribed for purposes other than the common cold. To consider individual drugs, fosfomycin is an antibiotic generally used for urinary tract infections and it doesn’t have indication for bacterial pneumonia in Japan. Chloromycetin and sulfamethoxazole-trimethoprim can cause serious side effects and are unlikely to be prescribed for common colds; 3) those receiving antibiotics over 14 days to avoid chronic sinusitis or other chronic diseases (n = 1190); 4) those who had diseases of the external ear (H60-62), middle ear, and mastoid (H65-75) within 3 months or infectious gastroenteritis and colitis or unspecified (A09) and other bacterial intestinal infections (A04) in the same month diagnosed with common cold (J00) (n = 5169); or 5) those who switched antibiotics (n = 1113), because patients whose doctors made the decision to switch antibiotics were excluded from the study because such patients can have an underlying medical condition that required chronic antibiotic treatment and may not have been prescribed antibiotics for cold symptoms. In 21,670 patients, we excluded family members (n = 3011). Finally, we analyzed 18,659 working-age worker patients with common cold.

### Study endpoints and definitions

Data of eligible patients were used for assessing the primary and secondary endpoints. The primary endpoint was the proportion of co-administration of common cold drugs and antibiotics in working-age population workers. The secondary endpoint was predictive factors of prescribing antibiotics in patients with common cold.

### Statistical analysis

Univariate analyses (Student’s t-test, Mann–Whitney U-test, and chi-square test) of the groups for “with antibiotics” and “without antibiotics” were performed using patient demographics of 18,659 patients.

We followed standard methods to estimate sample sizes for multiple logistic regression. At least 10 outcomes were needed for each independent variable to avoid overfitting. We analyzed associations between the simultaneous prescription of common cold drugs and antibiotics with potential predictors such as sex (male or female), age (year), hospital or clinic scale with number of beds diagnosed with common cold, and comorbidity for neoplasms (C00-D49), chronic lower respiratory diseases (J40-J47), hypertensive diseases (I10-I15), atrial fibrillation and flutter (I48), disorders of lipoprotein metabolism and other lipidemias (E78), inflammatory polyarthropathies (M05-M14), diabetes mellitus (E10-14), and non-infectious enteritis and colitis (K50-K52).

Data are expressed as medians with ranges or mean ± standard deviations. Data analysis was performed using JMP 15® (SAS Institute Inc., Cary, NC, USA) and Bell curve for Excel® (Social Survey Research Information Co. Ltd., Tokyo, Japan).

### Ethics approval and consent to participate

The commercially available database of JMDC. Inc. used in this study is anonymized processed information based on Japan's Personal Information Protection Law, and individual informed consent is not required for provision and use. In addition, according to the ethical guidelines for clinical research in Japan, these researches using anonymized processed information are not required to be reviewed by an ethical review committee.

## Data Availability

JMDC (Japan Medical Data Center, Tokyo, Japan) claims database can be commercially available to access from JMDC inc. Data sharing policy is available from JMDC’s web site (https://www.jmdc.co.jp/en/bigdata), and database created by JMDC was detailed by Nagai K et al.^18^. Our research data are not available due to the restriction by the JMDC policy to provide 3rd party from researchers in other objectives.
